# Electrophilic Susceptibility of Graphene Quantum Dots: Hypochlorous versus Hypobromous Acids—Experimental and Theoretical Study

**DOI:** 10.1021/acsomega.5c01500

**Published:** 2025-04-11

**Authors:** Guilherme Justiniano Mizumoto, Nelson Henrique Morgon, Aguinaldo Robinson de Souza, Valdecir Farias Ximenes

**Affiliations:** 1 Departamento de Química, Universidade Estadual Paulista Júlio de Mesquita Filho (UNESP), Bauru, São Paulo 17033-360, Brazil; 2 Departamento de Físico-Química, Instituto de Química, Universidade Estadual de Campinas, Campinas, São Paulo 13083-861, Brazil

## Abstract

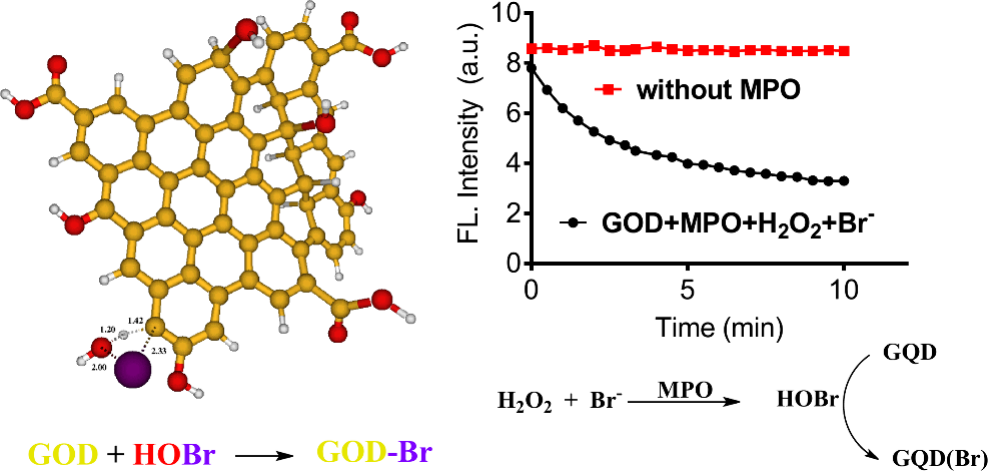

Graphene quantum dots (GQDs) are water-soluble, are biocompatible, and exhibit low toxicity. These properties, along with their adjustable and efficient fluorescent emission, make GQDs valuable for biological applications, particularly as spectroscopic nanosensors. In this context, GQDs have been utilized to detect hypochlorous acid (HOCl). While HOCl is a well-known synthetic disinfectant, it is also naturally produced by the enzyme myeloperoxidase (MPO) in mammals. This heme-peroxidase also catalyzes the production of hypobromous acid (HOBr), a more potent halogenating agent. In our study, we compared the reactivity of HOCl and HOBr with GQDs. By monitoring the fluorescence bleaching of the GQDs, we demonstrated that HOBr is more reactive than HOCl. The increased reactivity was attributed to HOBr’s higher electrophilicity. The electrophilic nature of the reaction was further confirmed by introducing nicotine as a chlorination catalyst. Anisole did not inhibit the electrophilic attack, confirming the high reactivity of GODs with HOBr. The enzyme MPO was used to generate HOBr through oxidation of Br^–^ by H_2_O_2_. Thus, the enzymatic activity of MPO could be monitored by GQDs’ fluorescence bleaching, and the efficiency of MPO inhibitors could be evaluated. We applied differential function theory (DFT) methodologies to support our experimental findings, proposing a transition state for the electrophilic attack. Consistent with our experimental results, the energetic barrier for the reaction with HOBr was lower than that for HOCl. Overall, our results indicate the susceptibility of GQDs to electrophilic attacks by hypohalous acids and highlight new opportunities for biological applications.

## 1 Introduction

Graphene-based nanomaterials are two-dimensional (2D) atomic crystals made up of sp2-hybridized carbon atoms. One type of these materials is graphene quantum dots (GQDs), which are small fragments of graphene, typically less than 10 nm in size, consisting of one to a few layers of graphene.^1−4^ Due to its very small size, these DOTs are regarded as zero-dimensional nanomaterials.^1−3^ GQDs are water-soluble, biocompatible, and usually low-toxicity. These properties, along with their adjustable and efficient fluorescent emission, make them useful for biological applications such as spectroscopic nanosensors in a myriad of fluorescence-based detection techniques.^2,5^ This central feature of GQDs is related to the extended sp2-hybridized carbon structure, doping effects, and the presence of functional groups such as carboxylates, hydroxyl, epoxy, etc., which alter these materials’ excitation and emission.^2,4,5^ Furthermore, graphene quantum dots (GQDs) exhibit a size effect known as quantum confinement, which significantly influences their spectroscopic properties.^2,4,5^ In this sense, the fluorescence quantum yield of GQDs can be altered by analytes that react or interact via intermolecular forces. Based on these properties, numerous analytical methods have been developed to determine metals, drugs, antioxidants, etc.^6^ Recent studies have shown the application of boron and sulfur codoped graphene quantum dots (BS-GQDs) to analyze ibuprofen. In this research, a ground-state complex forms between BS-GQDs and ibuprofen, resulting in fluorescence quenching.^7^ Determining the concentration of hydrogen peroxide (H_2_O_2_) in cancer cells through fluorescence bleaching of GQDs synthesized using tryptophan.^8^ Evaluation of fish freshness using nitrogen-doped GQDs. In this work, the presence of ammonia leads to fluorescence quenching via photoinduced electron transfer.^9^

As suggested by the title, among the uses of GODs, the present work is particularly interested in detecting hypochlorous acid (HOCl), one of the applications of GQDs. The reported applications have a background in the reactivity and intrinsic fluorescence of GQDs, which enables the spectroscopic detection of HOCl in wastewater and biological mediums. For example, Golubewa et al. developed an assay that detects the production of hypochlorous acid (HOCl) by human neutrophils through the bleaching of GQDs’ green fluorescence.^10^ Parthiban et al. described the synthesis and application of bright blue fluorescent multifunctional carbon dots, reporting a detection limit of 3.38 nM of HOCl in deionized water.^11^ Similarly, Wang and his collaborators successfully utilized nitrogen-doped carbon dots to detect HOCl in tap and river water, achieving a detection limit as low as 1 nM.^12^ Other relevant works include: ″Selective determination of free dissolved chlorine using nitrogen-doped carbon dots as a fluorescent probe″.^13^ ″Carbon-based electrochemical free chlorine sensors″,^14^ and ″A hybrid system for the quantitative fluorescent determination of total antioxidant capacity″.^15^ It is important to highlight that the assays reported in these studies are based on the fluorescence decay resulting from the chemical modification of graphene quantum dots (GQDs) due to a reaction with hypochlorous acid (HOCl). This chemical reaction can be enhanced by using hypobromous acid (HOBr), as will be demonstrated.

Besides being a well-known synthetic disinfectant, HOCl is also naturally produced in mammalians. This occurs when chloride ions (Cl^–^) are oxidized by H_2_O_2_ in a reaction catalyzed by the heme protein myeloperoxidase (MPO).^16−19^ This enzyme is highly expressed in neutrophilic polymorphonuclear leukocytes (PMNs), reaching 5% of the cell’s dry weight.^20,21^ This chemical machinery’s physiological role is to act as an antimicrobial agent, protecting the human body from infections. In other words, the endogenous production of HOCl is an essential part of the innate immune system.^16−18,22,23^ However, there is also a harmful side effect because strong evidence correlates MPO and biomarkers of MPO-catalyzed oxidations with inflammatory conditions such as coronary artery diseases,^24^ cystic fibrosis,^25^ neurodegeneration,^26^ carcinogenesis and many others.^27^ This is because, in chronic inflammation, the uncontrolled production of HOCl has deleterious effects on the human body and is linked to the onset and progression of these pathologies.^28,29^

MPO can also catalyze the oxidation of bromide (Br^–^) to hypobromous acid (HOBr). At first glance, this reaction may seem less significant, especially considering that the plasma concentration of Br^–^ (20–100 μM) is approximately 1000 times lower than that of Cl^–^, which is present at around 100 mM in blood plasma.^30,31^ However, the formation of HOBr still occurs, and its involvement in inflammatory diseases has been widely demonstrated.^32−35^ In addition, the equivalent peroxidase known as eosinophil peroxidase (EPO) has Br^–^ as the preferential substrate compared to Cl^–.^^36^ EPO is expressed in eosinophil leukocytes, and the production of HOBr by these cells is well-documented, as well as the deleterious effect of HOBr.^37−39^

As stated, GQDs have been used as fluorescent sensors to detect and quantify HOCl in different matrices. HOBr is also an endogenously halogenating species that is relevant to tissue damage. Based on these well-established experimental data, it would be natural to evaluate the reactivity of GQDs with HOBr, which, as far as we know, has yet to be reported. In addition, GQDs have been described as a material susceptible to electrophilic attack, which is a significant chemical property of HOBr compared to HOCl.^40−42^ Thus, this work aimed to evaluate the reactivity of GQDs with HOBr and the experimental and theoretical aspects of the reaction. As will be shown, we obtained robust evidence of HOBr’s increased reactivity with GQDs compared to HOCl.

## 2 Experimental Section

### 2.1 Chemicals, Enzymes, and Solutions

Graphene quantum dots (10 mg/mL aqueous solution, N°900708), 5-fluorotryptamine, (−)-nicotine hydrogen tartrate salt, hypochlorous acid (HOCl) (12–15%), sodium bromide (NaBr), and hydrogen peroxide (H_2_O_2_) (30%) were purchased from Sigma-Aldrich Chemical Co. (St. Louis, MO, USA). Myeloperoxidase (MPO) (EC 1.11.1.7) was purchased from Planta Natural Products (Vienna, Austria). The studies were conducted in 10 mM buffered phosphate buffer (PBS) at pH 7.4 and 25 °C. Stock solutions of HOCl (100 mM) were prepared daily by diluting the 12% commercial solution, and the concentration was determined spectrophotometrically after dilution in 0.01 M NaOH, pH 12 (ε_292 nm_ = 378 M^–1^cm^–1^).^43^ Stock solutions of hypobromous acid (HOBr, 100 mM) were prepared by combining 100 mM HOCl and 200 mM NaBr in water.^36^ Stock solutions of H_2_O_2_ (100 mM) were prepared by diluting the commercial 30% solution in water, and the concentration was determined spectrophotometrically (ε_240 nm_ = 43.6 M^–1^cm^–1^).^44^ Ultrapure Milli-Q water was used throughout the studies to prepare buffers and solutions.

### 2.2 Reactions of Hypohalous Acids with GQDs: General Procedure

The reactions of QGDs with HOCl and HOBr were monitored following the fluorescence and UV–vis absorption alteration. Unless otherwise stated, the reaction systems were constituted by GQDs (10 μg/mL), HOCl, or HOBr (200 μM) in PBS buffer, pH 7.4 at 25 °C. For the fluorescence studies, the experiments were performed using black flat bottom 96 well microplates. The reaction mixture was stirred and incubated for 5 min (orbital mixer). The fluorescence was recorded at an excitation of 345 nm and emission in the 375–450 nm range using a BioTek Synergy H1Multimode Reader (Agilent, Santa Clara, CA, United States). The absorbance spectra were measured using a PerkinElmer Lambda 35 UV–visible spectrophotometer (Shelton, CT, USA).

### 2.3 Enzymatic Generation of Hypohalous Acids

Hypohalous acids (HOCl and HOBr) were enzymatically generated via MPO-catalyzed oxidation of chloride and bromide by H_2_O_2._^45^ The reactions were conducted in PBS buffer, pH 7.4, which, besides pH control, acted as a source of chloride ions. Unless otherwise stated, the reaction medium was constituted by GQDs 10 mg/mL, NaBr 20 mM, MPO 20 nM, and H_2_O_2_ 50 μM. The reactions were triggered by adding H_2_O_2_. The reactions were monitored at 345/450 nm at 25 °C. The reactions were conducted in microplates, as presented in section 2.2.

### 2.4 Computational Methodology

**Figure 1:**
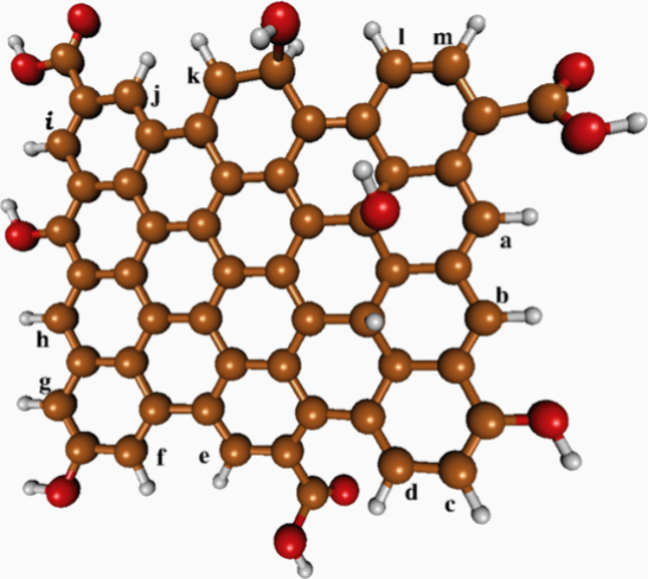
Molecular structure of GQDs and the theoretically evaluated positions (carbons “a” to “m”) for electrophilic attack by HOX (X = Cl or Br). The molecular structure was reprinted from PubChem (CID 146000141 https:// https://pubchem.ncbi.nlm.nih.gov/compound/146000141).

The computational methodology employed in studying the mechanism consisted of the following stages: GQDs + HOX → GQD-X + H_2_O, X = Cl or Br. Initially, the molecular geometries of the reacting species, GQDs and HOX, were optimized using the semiempirical model PM7. This optimization aimed to determine the minimum energy configurations for both reactants and products (GQDs-X and H_2_O). Subsequently, harmonic vibrational frequency calculations were performed to characterize the stationary points, confirming their energetic minimum nature by identifying positive vibrational frequencies. The descriptor employed in the reactivity study was the activation energy barrier. The transition state (TS) identification was conducted by considering the potential center of attack of HOX on carbon atoms adjacent to the −COOH and −OH groups of GQD, as depicted in Figure 1 (positions ″a″ to ″m″). The molecular structure of the GQDs was used as described.^46^ Characterizing the identified TS involved harmonic vibrational frequency calculations to verify the presence of a single imaginary frequency, a hallmark of saddle points. Furthermore, the intrinsic reaction coordinate (IRC) was analyzed to confirm the connectivity between reactants and products via the minimum energy path. The IRC was computed using 100 points, a stepwise of 10 (the size of each step taken along the reaction path), and the local quadratic approximation (LQA) method. The IRC is the path that the molecule follows when passing from the transition state to the reactants or products, following the path of least energy, and the LQA method is both accurate - due to the use of curvature information, and efficient - requiring only one energy and one gradient calculation per step, and is, therefore, a good choice for the micro iterative IRC procedure. All calculations were performed with the PM7 semiempirical method employing the Gaussian16 software package.^47−49^

## 3 Results and Discussion

### 3.1 Reactivity of GQDs: HOCl versus HOBr

To assess the reactivity of HOCl and HOBr with GQDs, we employed a commercial 10 mg/mL aqueous solution of the nanoparticles (Sigma-Aldrich, N°900726). The material has been characterized and has a topographic height of 1.0–2.0 nm, particle size <5 nm, and the following spectroscopic properties: λ_ex_ 350 nm, λ_em_ 445 nm ± 10 nm, and quantum yield ≥ 20%. Aiming posterior enzymatic application, the reactions were conducted in PBS buffer at pH 7.4.

**Figure 2:**
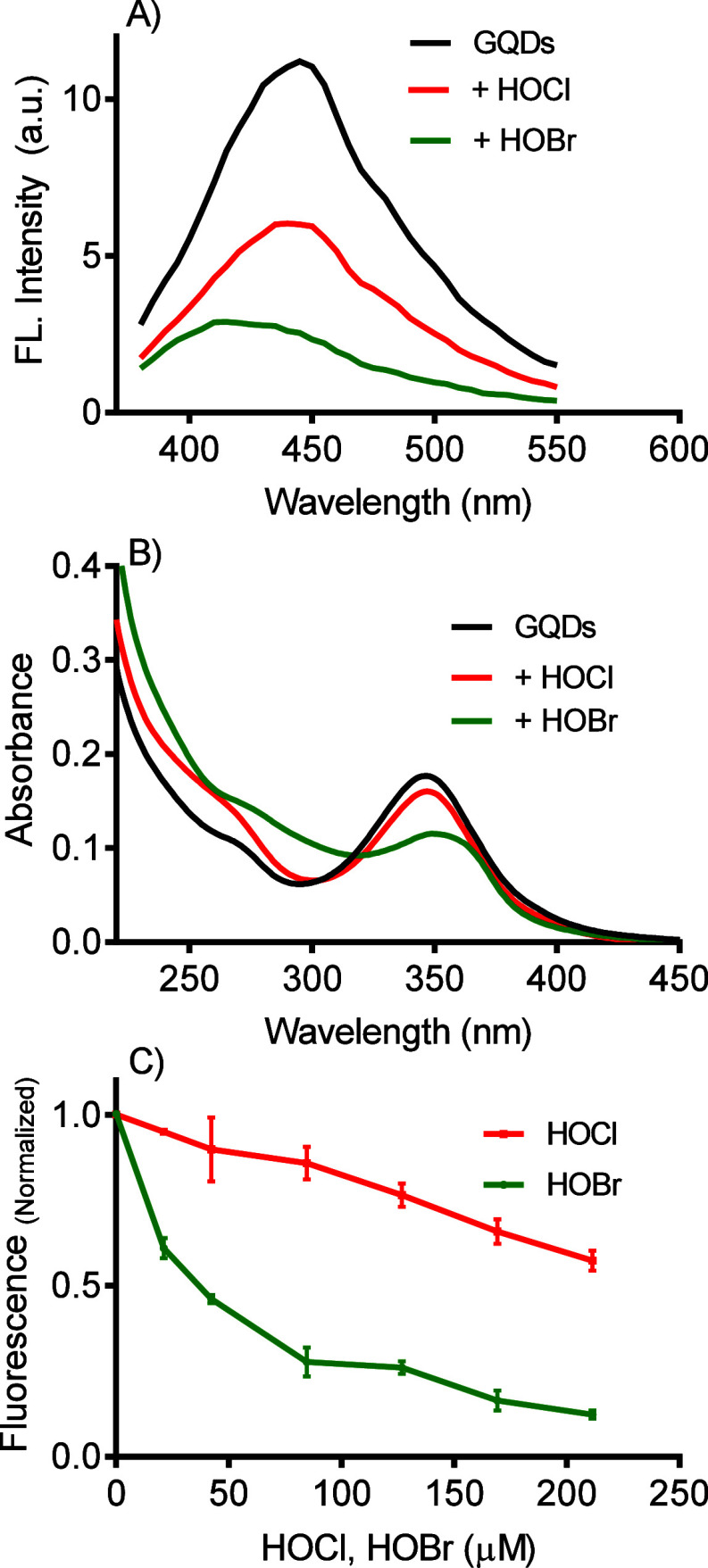
Spectral alteration of GQDs due to reaction with hypohalous acids: HOCl versus HOBr. **A**) Fluorescence decay, **B**) UV–vis alteration, and **C**) Concentration-dependent fluorescence decay (mean and SD of triplicates). Excitation and emission at 345/450 nm. Experimental conditions: GQDs 10 μg/mL, HOCl, and HOBr 200 μM (panel A and B), PBS pH 7.4, 25 °C.

We found that HOBr is a significantly more effective bleaching agent for graphene quantum dots (GQDs) fluorescence than HOCl, as shown in Figure 2A. Specifically, adding 200 μM of HOBr was sufficient to completely deplete the intrinsic fluorescence of 10 μg/mL GQDs. In these experiments, the remaining, blue-shifted fluorescence band did not change even when higher concentrations of HOBr or HOCl were applied, suggesting that the reaction product also possesses fluorescence properties. The greater reactivity of HOBr was further demonstrated by recording the UV–vis absorption spectra before and after the addition of the hypohalous acids, as shown in Figure 2B. The absorption band of GQDs centered at 350 nm, decreased and shifted upon adding HOBr. In contrast, the changes observed with HOCl were less pronounced, consistent with the fluorescence results. Figure 2C presents the titration of the GQDs solution with both acids, confirming the previous findings and emphasizing the higher reactivity of HOBr.^40,50^

As stated above, HOBr’s greater reactivity with GQDs than HOCl must be due to its higher electrophilic strength. This is a crucial aspect of our research because GQDs are, despite oxygenated functional groups, essentially composed of sheets of sp2-hybridized carbon atoms, i.e., a polycyclic aromatic hydrocarbon. Therefore, GQDs should be as susceptible to electrophilic attack as other aromatic compounds. Corroborant with our proposal, experimental evidence of the susceptibility to electrophilic attack has been reported. For instance, the electrophilic fluorination of graphene oxide^51^ and electrophilic alkylation.^52^ Hypohalous acids are typical agents for electrophilic halogenation of halogenate electron-rich arenes without requiring additional catalysts.^53^ The higher reactivity of HOBr as an electrophilic agent for halogenation has also been demonstrated in reactions involved in water treatment.^50^ The increased reactivity of HOBr compared to HOCl is due to the involvement of the intermediate halonium ion in electrophilic halogenation reactions mediated by these species. This is due to the lower electronegativity and higher polarizability of the bromine atom, which promote the formation of a bromonium ion (Br^+^), the intermediate electrophilic species involved in the halogenation of arenes.^40,54^ Many examples in the literature demonstrate the HOBr’s higher reactivity compared to HOCl.^42,45,50,55,56^ Given the well-established chemical property of HOBr, it is reasonable to assume that the susceptibility of GQDs to electrophilic attack is central to explaining our findings.

In order to strengthen our proposal, we conducted an experiment to evaluate the reaction between HOBr and GQDs with and without anisole. Anisole is an aromatic compound containing an electron-donating substituent (methoxy group), which makes it vulnerable to the electrophilic attack of HOBr but not enough to react with HOCl.^40^ It is important to note that only activated aromatic compounds are susceptible to electrophilic attack by HOBr. For example, acetophenone, which has a ring-deactivating electron-withdrawing substituent (acetyl group), is unreactive.^40^

**Figure 3:**
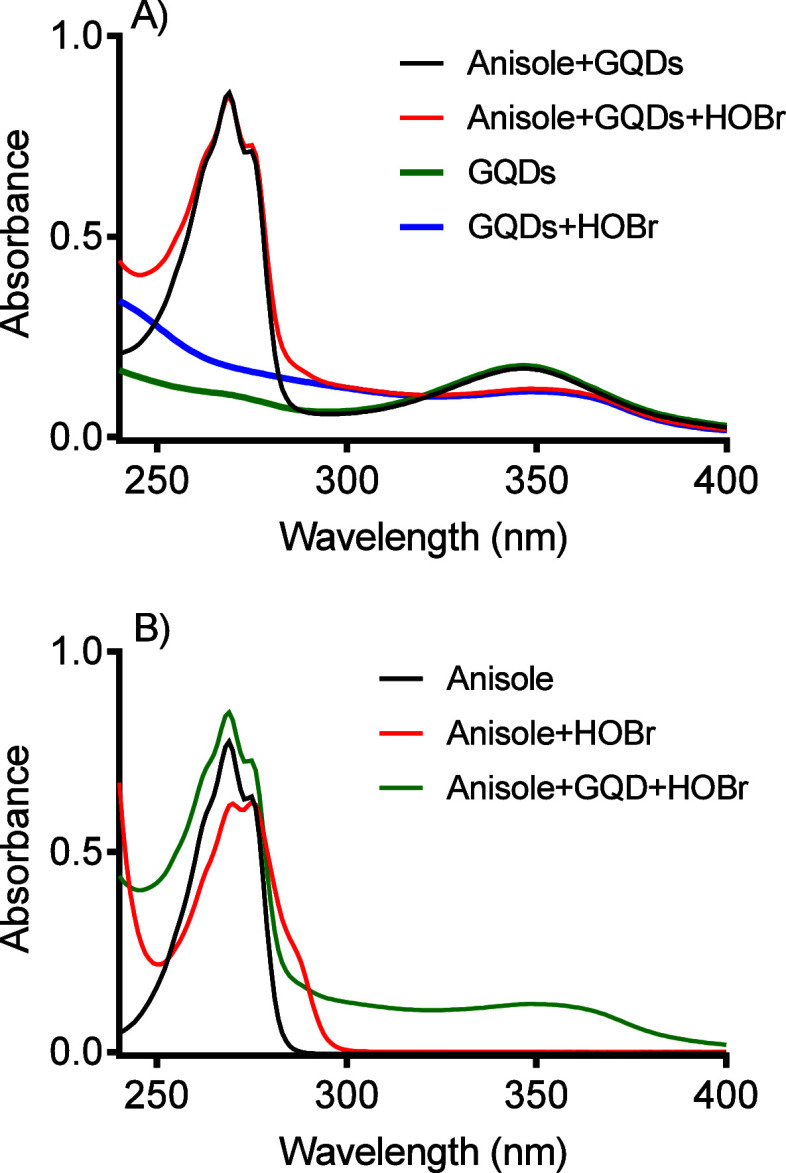
Competition between GQDs and anisole by HOBr. **A**) The effect of anisole on GQDs alteration due to HOBr. The presence of anisole could not impede the alteration in the GQDs′ UV–vis band at 350 nm. **B**) The effect of GQDs on anisole alteration due to HOBr. The presence of GQDs impeded the alteration in the anisoles UV–vis band at 280 nm. Experimental conditions: GQDs 10 μg/mL, HOBr 400 μM, anisole 500 μM, PBS pH 7.4, 25 °C.

According to the results in Figure 3, anisole did not hinder the reaction of HOBr with GQDs. As shown, the absorption band at 350 nm decreased equally in the presence and absence of anisole (Figure 3A). Additionally, the absorption band of anisole (280 nm) remained unchanged, confirming that GQDs reacted preferentially. In another experimental approach, we observed the reaction of anisole with HOBr with and without GQDs. Figure 3B displays the change in the anisole UV–vis band due to adding HOBr, confirming that this aromatic compound is susceptible to HOBr.^40^ However, in the presence of the GQDs, the reaction was inhibited, confirming the higher reactivity of the nanomaterial. In short, considering that only electron-rich activated aromatic compounds are susceptible to electrophilic attack by hypohalous acids,^53^ the higher reactivity of GQDs compared to anisole strongly indicates their significant susceptibility to electrophilic-initiated reactions.

**Scheme 1:**
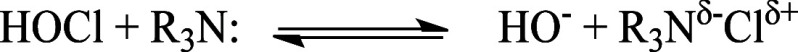
Generation of Quaternary Chloramonium Ions Is Responsible for the Increased Electrophilicity of HOCl

To validate our proposal regarding the significance of GQDs’ electrophilic susceptibility, we utilized nicotine as a catalyst in the reaction with HOCl. The proposal is based on the known role of tertiary amines as catalysts for the chlorination of alkenes and arenes by HOCl. The mechanism is based on the involvement of the tertiary amine’s electrophilic intermediate quaternary chloramonium ions.^57,58^Scheme 1 illustrates the mechanism of tertiary amines as catalysts of HOCl chlorination.^57,58^ Nicotine, trimethylamine, and quinine are examples of tertiary amines used in this proposal.^57,59^

**Figure 4:**
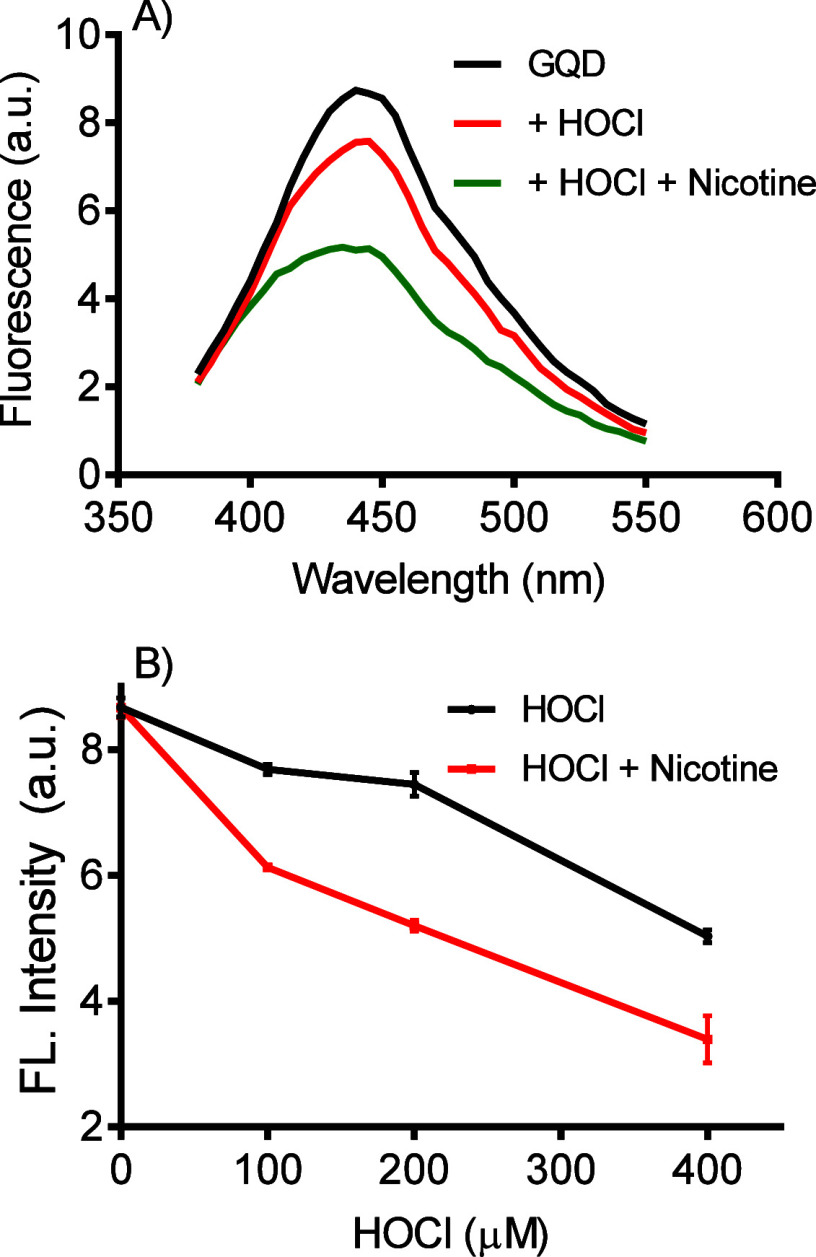
Nicotine as a catalyst for HOCl-mediated chlorination of GQDs. **A**) GQDs fluorescence bleaching in the presence and absence of nicotine. Reaction condition: GQDs 10 μg/mL, HOCl 200 μM and nicotine 100 μM in PBS pH 7.4, 25 °C. **B**) Concentration-dependent effect of HOCl in the presence and absence of nicotine. The results are the mean and SD of triplicates.

Corroborant with these expectations, adding nicotine improved the fluorescence bleaching of GQDs by HOCl (Figure 4). Considering the mentioned features of nicotine as a catalyst of the electrophilic role of HOCl, this finding reinforces the proposal of GQD’s electrophilic susceptibility.

### 3.2 Detection of Myeloperoxidase: The Role of Bromide

**Scheme 2:**
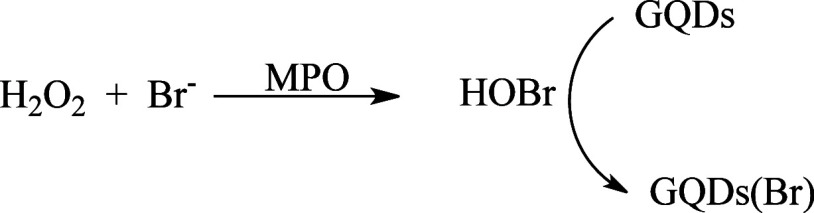
Proposed Chemical Equation for MPO-Catalyzed Generation of HOBr and the Use of GQDs as a Fluorescent Probe to Monitor the Reaction Halogenation leads to the bleaching of fluorescence in GQDs.

**Figure 5:**
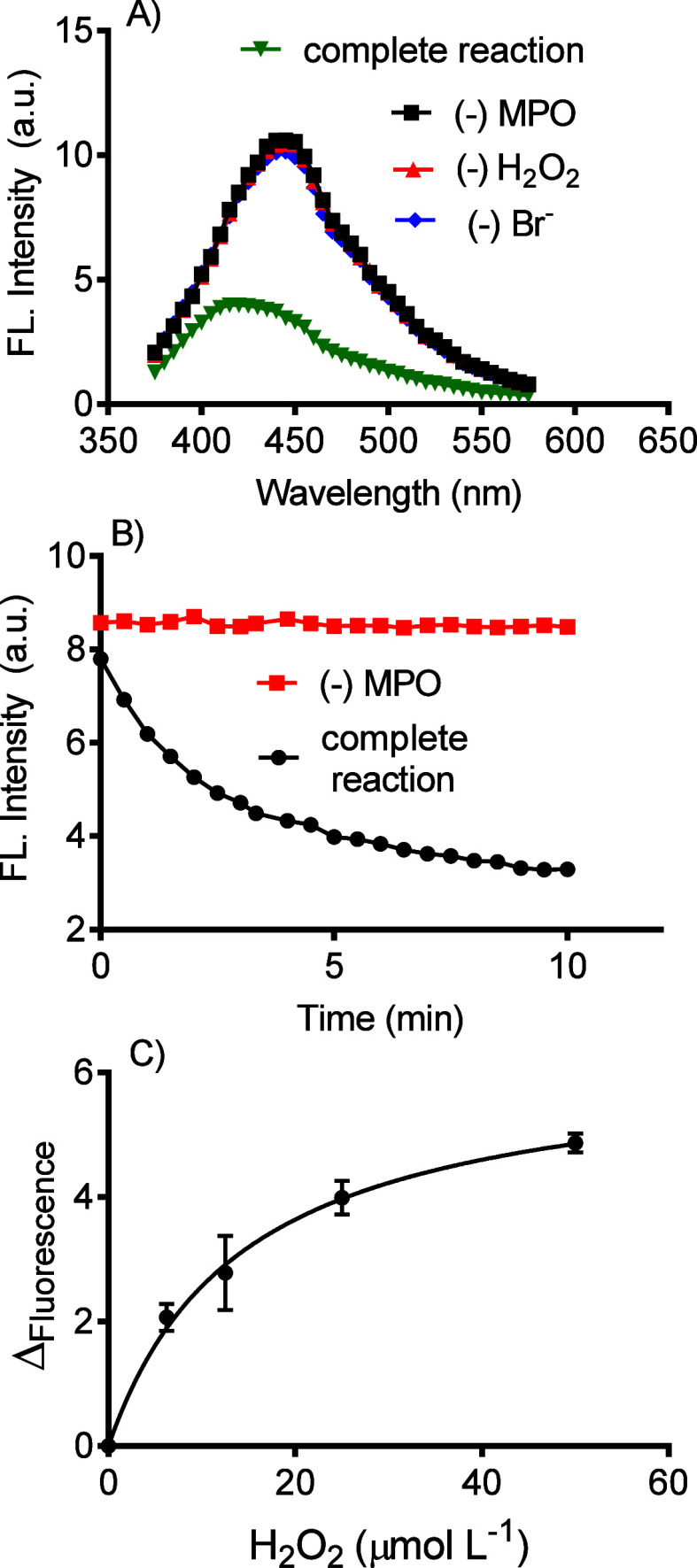
Myeloperoxidase-catalyzed generation of hypohalous acids and the effect on GQDs. **A**) GQDs fluorescence bleaching for the complete reaction system and in the absence of the enzyme and reactants. Complete reaction system: MPO 20 nM, GQDs 10 μg/mL, H_2_O_2_ 50 μM, Br^–^ 20 mM in PBS pH 7.4, 25 °C. **B**) Kinetic profile of fluorescence bleaching in the presence and absence of MPO. **C**) Concentration-dependent effect of H_2_O_2_ on oxidant for generation of HOBr. Reaction condition: MPO 8 nM, GQDs 10 μg/mL, Br^–^ 20 mM in PBS pH 7.4, 25 °C. The results are the means and SD of triplicates.

Detecting HOCl in biological media is essential because it is naturally produced in the body and plays a role in the innate immune system. In this regard, neutrophil cells are the primary source of HOCl, which primarily functions to destroy invading pathogens but is also associated with chronic inflammatory diseases.^23,29^ Endogenously, the oxidation of Br^–^ to HOBr is also catalyzed by MPO. The rate constant for the reaction between the redox active form of MPO (compound I) and Br^–^ is about 100-fold higher than Cl^–.^^60^ Therefore, despite the low plasma level of Br^–,^^30,31^ the formation of HOBr still occurs, and its involvement in inflammatory diseases has been widely demonstrated, as stated above. Thus, the next task was to evaluate the reaction in the presence of MPO, where pure HOCl and HOBr were substituted by Cl^–^, Br^–^, and H_2_O_2_. The results depicted in Figure 5A confirmed our expectation once the enzymatically generated HOBr led to GQDs fluorescence bleaching. As expected, the absence of Br^–^, H_2_O_2_, or MPO impeded the reaction, confirming the enzymatic generation of HOBr. Confirming the previous experiment with pure HOCl, the fluorescence bleaching was minimal without Br^–^. Figure 5B shows the reaction’s kinetic profile and the catalyst’s fundamental role of MPO. Figure 5C shows the dependence of H_2_O_2_ as an oxidant. It is noteworthy that H_2_O_2_ was not reactive with GQDs without MPO or Br^–^. Scheme 2 shows the chemical equation for the enzymatic generation of HOBr and the application of GQDs to monitor the reaction.

**Figure 6:**
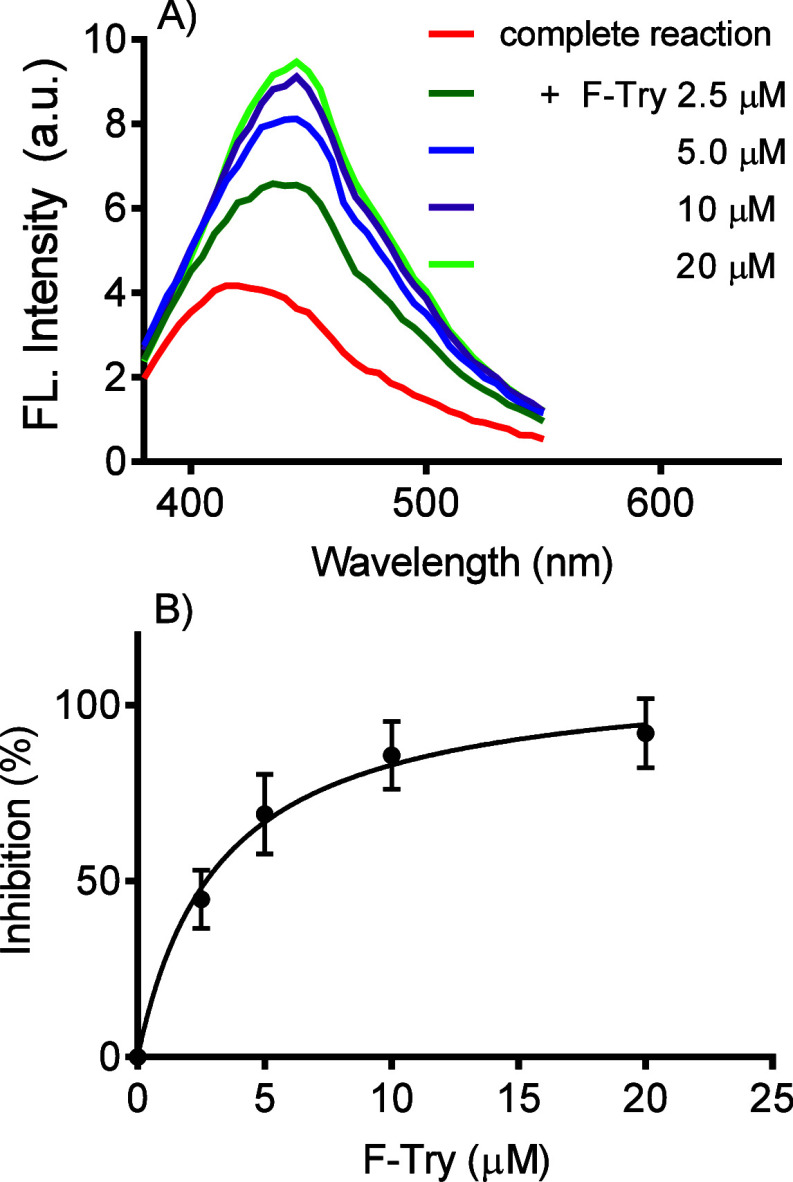
Evaluation of 5-fluorotryptamine (F-Try) as MPO inhibitor. A) GQDs fluorescence bleaching for the complete reaction system and the effect of F-try. B) Inhibition of HOBr production measured by GQDs fluorescence decay. Reaction condition: Complete reaction: MPO 20 nM, H_2_O_2_ 50 mM, GQDs 10 mg/mL, Br^–^ 20 mM in PBS pH 7.4, 25 °C.

A specific inhibitor of the enzyme’s halogenating activity was assessed to validate our results and the reliance on MPO as a catalyst for generating HOBr. The compound 5-fluorotryptamine (F-try) is an efficient and selective inhibitor of MPO, which blocks the enzyme’s redox cycle.^61^Figure 6A shows the effect of increasing concentrations of F-Try on fluorescence bleaching, and Figure 6B shows the concentration-dependent inhibition of MPO activity. This result confirmed the relationship between GQDs’ fluorescence bleaching and the enzymatic activity of MPO.

### 3.3 Theoretical Study

**Scheme 3:**
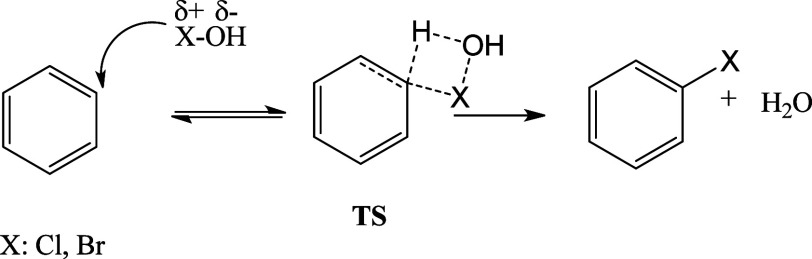
Reaction Mechanism and Transition State (TS) Were Evaluated Theoretically for the Reaction GODs + HOX1 The complete molecular structure of GQDs is shown in Figure 1.

The previous section provided substantial evidence of the electrophilic attack of hypohalous acids on GQDs. Additionally, we have demonstrated that HOBr is significantly more reactive than HOCl. To further validate these experimental findings, we conducted theoretical studies using density functional theory (DFT). The main objective was to assess the energetic profiles and transition states for the electrophilic attack on the aromatic rings of GQDs. It is worth mentioning that the theoretical study was conducted using a 2D graphene one-sheet model. This is not a real graphene-quantum dot, which usually comprises several sheets, a minimum of 7.^62^ However, given the resource-intensive nature of these calculations, expanding the system size beyond the current parameters was unfeasible with the available computational infrastructure. This trade-off between accuracy and scalability is inherent to first-principles studies.^63,64^Scheme 3 illustrates the proposed mechanism, and the transition state (TS) explored in the theoretical investigation. The molecular structure of the used GQDs is depicted in Figure 1.

Tables 1 (HOCl) and 2 (HOBr) present the total electronic energy, activation energy, and imaginary frequencies of the transition states for both GQD···(H)···(X)···OH systems at the potential attack positions (carbons) by HOX, as defined in Figure 1. The results presented here were obtained by considering the equilibrium geometries of all molecular species involved in the mechanism of the reaction GQD + HOX (where X = Cl or Br) leading to GOD-X + H_2_O. Calculations for the single-point energies and electronic properties were performed using the B3LYP/6–311++G(2d,p) level of theory, incorporating GD3BJ dispersion corrections. This approach employs the D3 version of Grimme’s dispersion method with Becke-Johnson damping, specified by B3LYP-D3(BJ)/6–311++G(2d,p)//PM7.^65^ As shown, the molecular system resulting from the attack of HOBr on the ″g″ carbon of GQD exhibits the lowest activation energy (72.24 kcal/mol). It is thus the most favored from the point of view of kinetic control.

**Table 1:** Reaction with HOCl: Total Electronic Energy, Activation Energy, and Imaginary Frequencies of the Transition States GQD···(H)···(Cl)···OH

position of attack in GQDs^a^	total electronic energy (hartree)	activation energy^b^ (kcal mol^–1^)	imaginary frequency (cm^–1^)
a	–3552.32495752	99.18	–1476.06
b	–3552.32778515	97.41	–1479.45
c	–3552.33719173	91.51	–1503.51
d	–3552.32861658	96.89	–1450.50
e	–3552.33014415	95.93	–1498.32
f	–3552.33625308	92.09	–1514.82
g	–3552.33988147	89.82	–1518.81
h	–3552.33570283	92.44	–1506.15
I	–3552.32749857	97.59	–1561.18
J	–3552.33152607	95.06	–1529.57
K	–3552.33777591	91.14	–1596.13
L	–3552.32655883	98.18	–1521.05
m	–3552.33200451	94.76	–1446.57

a The positions of attack on GQDs (a-m) by HOCl are defined in Figure 1.

b The energy of the reactants (GQD and HOCl) is equal to −3552.483023072 hartree.

**Figure 7:**
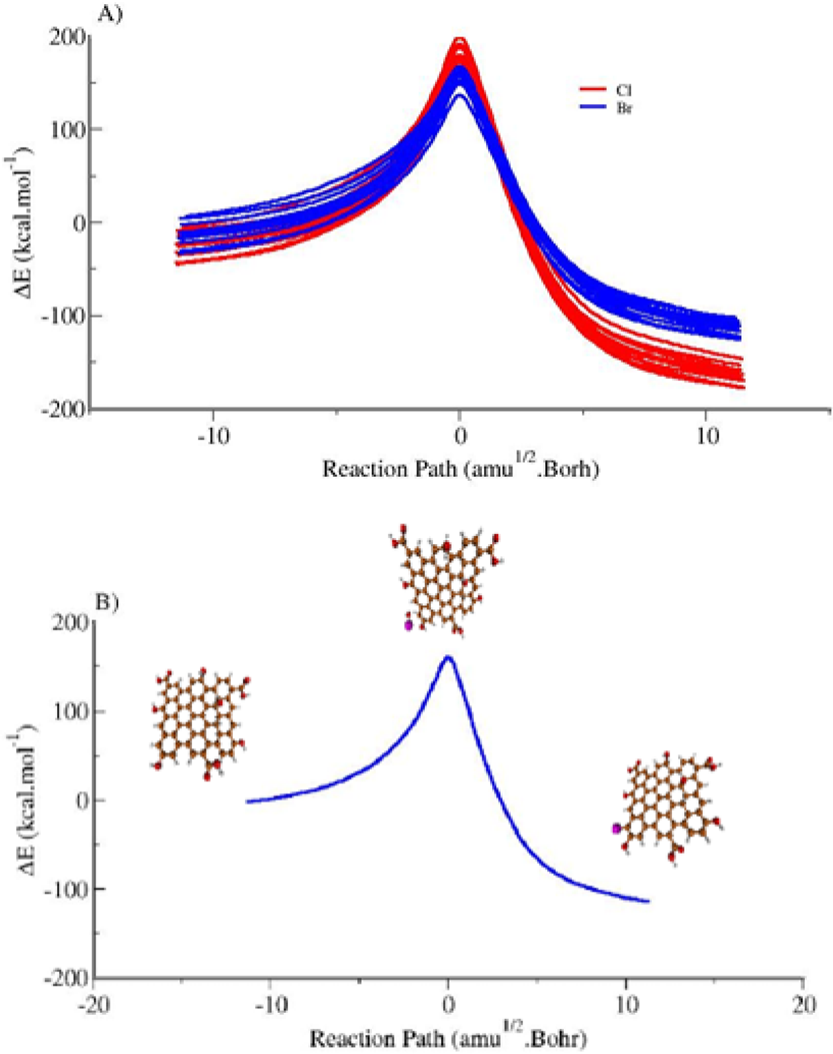
Intrinsic reaction coordinates (IRCs) for the reaction of GQDs with HOX (X = Cl or Br) to form GOD-X and H_2_O: (**A**) IRCs for both X = Cl and X = Br. (**B**) IRC for the reaction of HOBr at the ″g″ carbon of GQDs. All calculations were obtained at PM7 model.

Figure 7A displays all the IRCs associated with the transition states, with their corresponding imaginary frequencies listed in Tables 1 and 2. The imaginary frequencies, corresponding to the characterization of the transition state structures, were fully optimized.

**Table 2:** Reaction with HOBr: Total Electronic Energy, Activation Energy, and Imaginary Frequencies of the Transition States GQD···(H)···(Br)···OH

transition states^a^	total electronic energy (hartree)	activation energy^b^ (kcal mol^–1^)	imaginary frequency (cm^–1^)
a	–5666.28781907	80.49	–1315.76
b	–5666.29174549	78.03	–1336.25
c	–5666.29785262	74.20	–1364.36
d	–5666.29162643	78.10	–1320.17
e	–5666.29891589	73.53	–1385.11
f	–5666.29707439	74.68	–1375.26
g	–5666.30096237	72.24	–1375.00
h	–5666.29902957	73.46	–1371.54
i	–5666.29274680	77.40	–1416.73
j	–5666.29360593	76.86	–1401.98
k	–5666.29437300	76.38	–1436.07
l	–5666.28569410	81.83	–1360.07
m	–5666.29258525	77.50	–1314.38

a The positions of attack on GQD (a-m) by HOBr are defined in Figure 1.

b The energy of the reactants (GQD and HOBr) is equal to −5666.41609963 hartree.

The distinct lines in Figure 7A correspond to different reaction coordinates associated with the attacks of HOX species on the edge carbon atoms of the GQDs structure, as labeled ‘a’ to ‘m’ in Figure 1. The critical observation is that the activation barriers (or activation energies) for HOBr attacks are systematically lower, indicating a kinetic preference for these pathways. To visually distinguish these trends, we grouped the data using red for HOCl and blue HOBr (Figure 7A). Consistent with the experimental findings, it was observed that the transition states resulting from the attack of HOBr (represented by blue curves) have lower relative energies than those from the attack of HOCl (shown in red curves). Notably, the attack of HOBr at the ″g″ carbon yields the lowest energy barrier. Figure 7B presents the profile of the corresponding IRC separately from the others.

**Figure 8:**
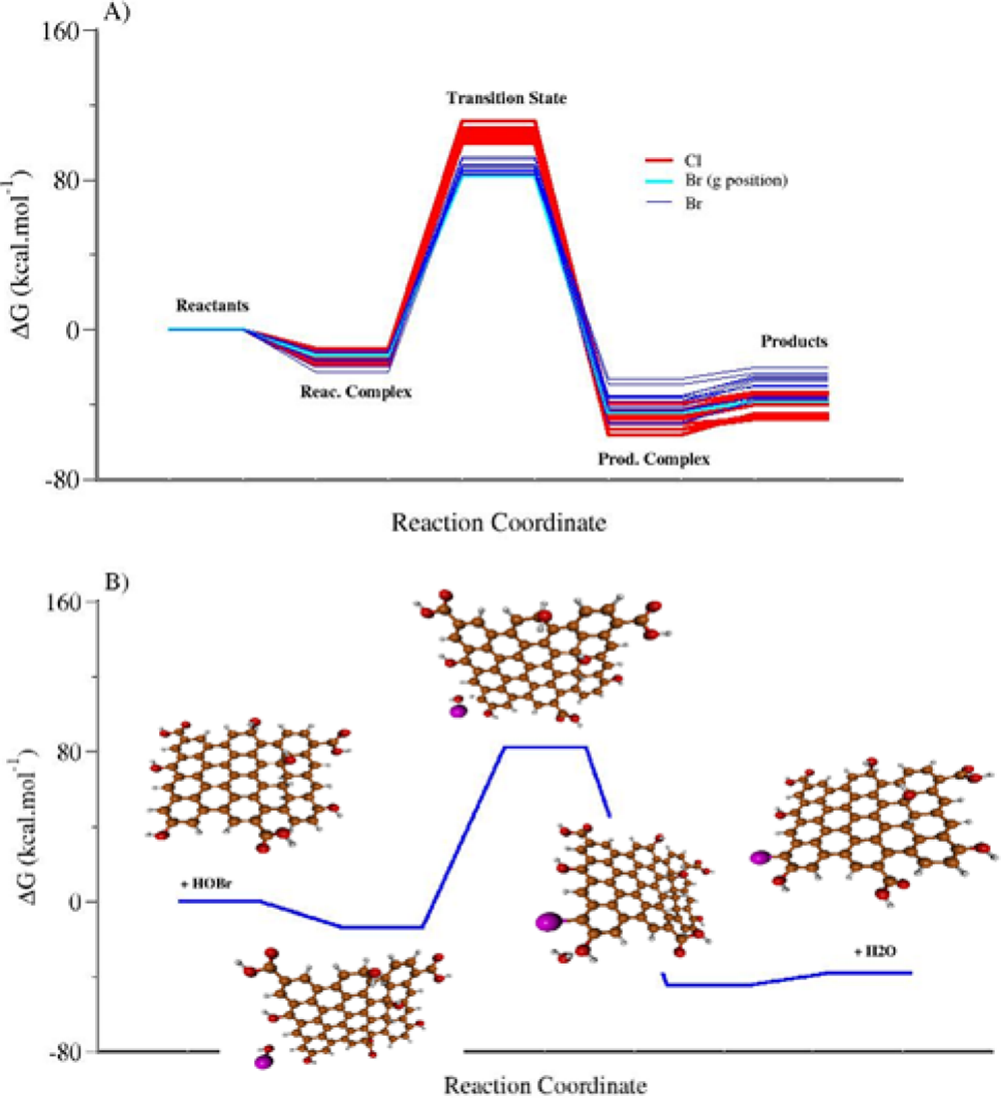
Reaction coordinate diagrams for the reaction of GQDs with HOX (X = Cl or Br) to form GOD-X and H_2_O: (A) reaction coordinates for attacks of HOX at all positions of GQDs (“a” to “m”); (B) reaction coordinate for the attack of HOBr at the ″g″ position of GQD. All calculations were performed at the B3LYP-D3(BJ)/6–311++G(2d,p)//PM7 level of theory.

**Figure 9:**
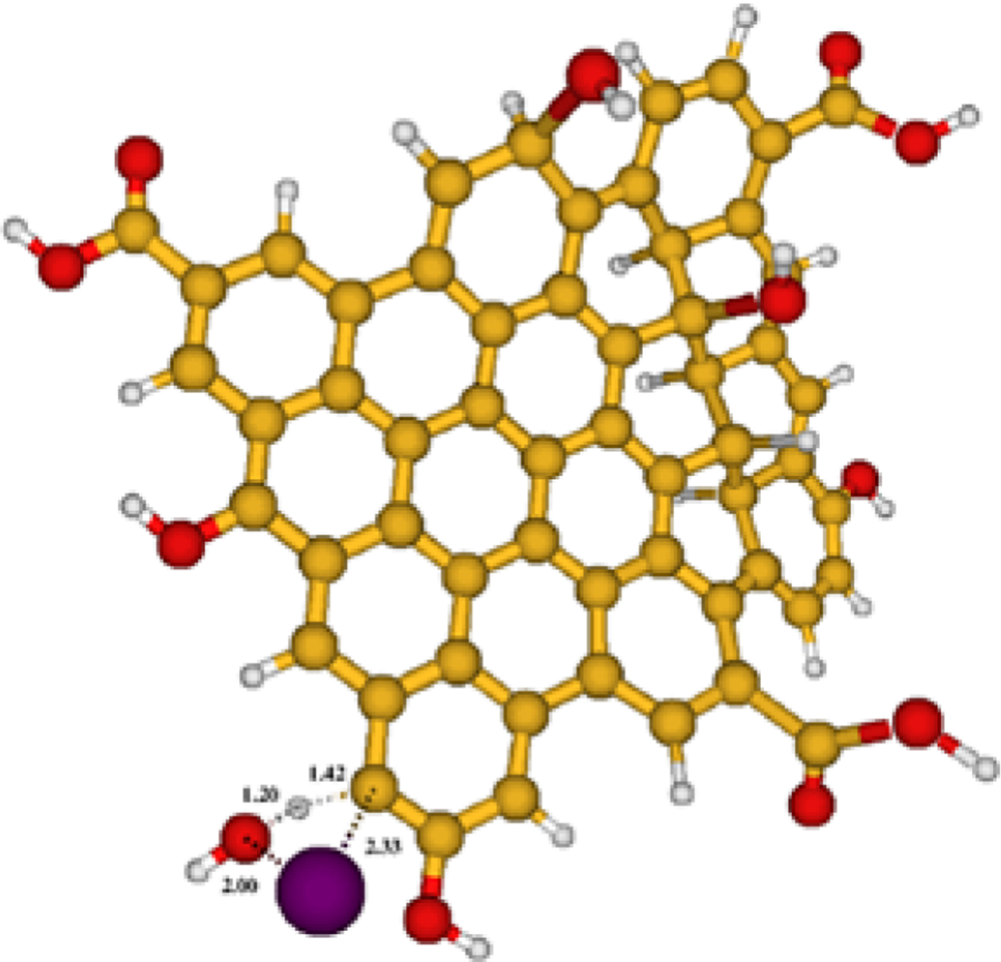
Structure of the proposed transition state for the reaction between HOBr and GQDs.

The reaction coordinates for the studied mechanism (GQD + HOX (X = Cl or Br) → GOD-X + H_2_O) are depicted in Figure 8. Figure 8A shows the respective complexes formed between the reactant molecules and the product molecules. All reactions have a slightly exothermic character. In Figure 8B, the reaction of HOBr at the ″g″ carbon is highlighted. It is observed that HOBr approaches GQD, the HOBr···GQD complex is formed, and the Br attacks the carbon at the ″g″ position. It is a mechanism that simultaneously involves the attack of Br to carbon, the abstraction of hydrogen bonded to this carbon, and the consequent formation of water before forming the H_2_O···Br-GQD complex. Figure 9 shows the molecular structure of the proposed transition state.

## 4 Conclusions

GQDs are vulnerable to electrophilic attack from HOCl and HOBr. The reaction with HOBr was found to be the most efficient due to its higher electrophilic strength. Additionally, nicotine serves as a catalyst that enhances the chlorination activity of HOCl, further improving reaction efficiency. The bleaching of GQDs fluorescence can be used to monitor the enzymatic production of HOBr, which occurs through the oxidation of Br^–^ by H_2_O_2_ with MPO as a catalyst. The decay in fluorescence can also be utilized to assess the effectiveness of MPO inhibitors. Theoretical studies support experimental results, showing lower energy levels for the transition states when using HOBr. These findings open a new venue for applying GQDs, which should be investigated further in developing new fluorescent probes.
